# Centennial CORLAS: Returning to Groningen, Where It All Began

**DOI:** 10.1055/s-0046-1825532

**Published:** 2026-08-14

**Authors:** Myrthe K.S. Hol, Antti A. Mäkitie, Sandro J. Stoeckli, Barbara Wollenberg, Pim van Dijk

**Affiliations:** 1 Department of Otorhinolaryngology/Head and Neck Surgery, University Medical Center Groningen, the Netherlands; 2 Research School of Behavioural and Cognitive Neurosciences, Graduate School of Medical Sciences, University of Groningen, the Netherlands; 3 Department of Otorhinolaryngology, Head and Neck Surgery, University of Helsinki and Helsinki University Hospital (HUS), Finland; 4 Department of Otorhinolaryngology, Head and Neck Surgery, HOCH Cantonal Hospital St. Gallen, Switzerland; 5 Department of Otorhinolaryngology, Head and Neck Surgery, University Hospital Rechts der Isar, Technical University of Munich, Germany

In 2026, the *Collegium Oto-Rhino-Laryngologicum Amicitiae Sacrum* (CORLAS) celebrates its centennial anniversary, a historic milestone within international academic otorhinolaryngology. The occasion is especially significant because the 100 ^th^ annual meeting will be held in Groningen, in the Netherlands, the city where the Collegium was founded in 1926 by Charles Emile Benjamins and Adriaan De Kleyn. One hundred years after its establishment, CORLAS returns to its place of origin, linking a ‘distinguished legacy’ with a promising future.

**Fig. 1.**
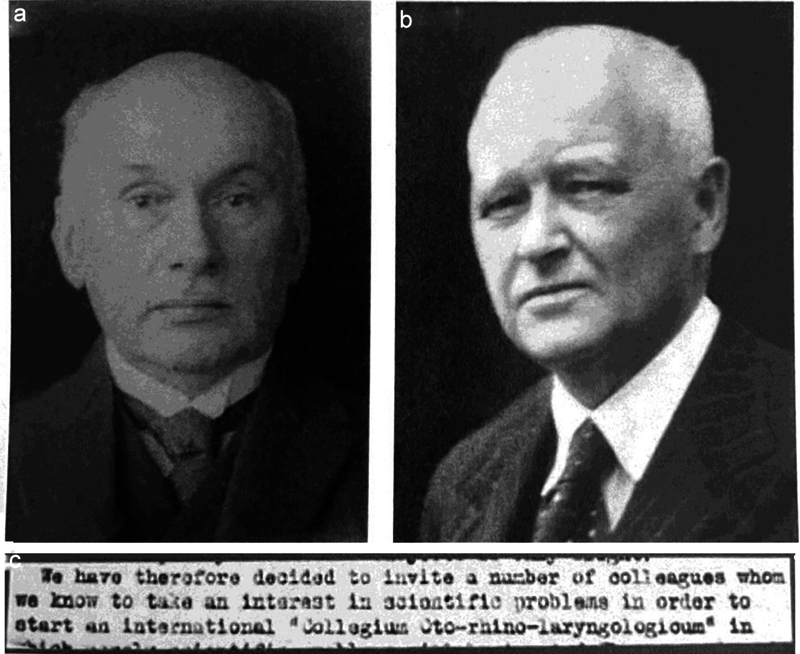
In the summer of 1926, ( **a** ) Prof. Charles E. Benjamins, from Groningen, and ( **b** ) Dr. Adriaan de Kleijn, from Utrecht, invited 58 colleagues from 13 different European countries ( **c** ) ‘whom we know to take an interest in scientific problems in order to start an international *Collegium Oto-rhino-laryngologicum* ’ 1 .

**Fig. 2.**
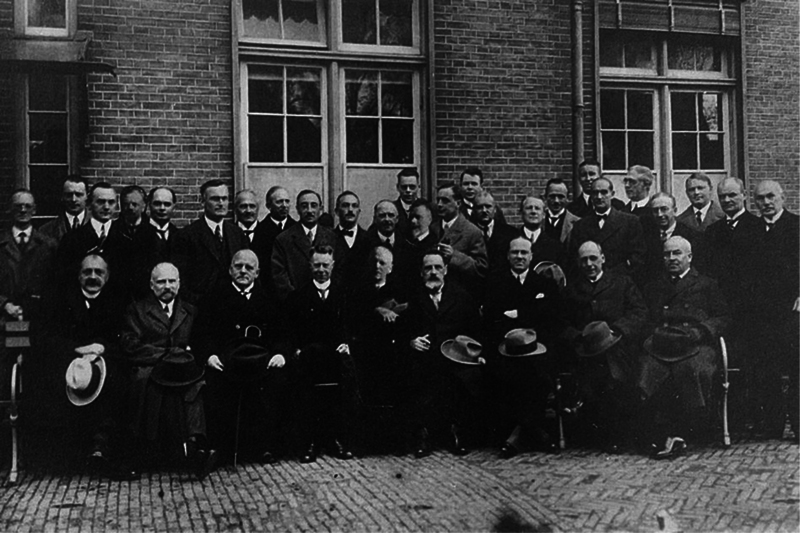
Group photograph taken in front of the building that housed the ENT clinic and where the scientific sessions of the founding meeting were held 2 .

The founding of CORLAS took place against the backdrop of a Europe still recovering from the divisions of the First World War 1 . At a time when scientific and personal relationships between former adversaries could not be taken for granted, Benjamins and De Kleyn sought to bring together leading academic otorhinolaryngologists from across Europe ( Figure 1 ). Their initiative was both ambitious and visionary: to promote scientific excellence through personal friendship and mutual respect. The first meeting was held in Groningen's historic Academy Building, establishing principles that continue to guide the Collegium today: scientific rigor, international collaboration, and *Amicitiae Sacrum* , sacred to friendship ( Figure 2 ).

Over the following century, CORLAS evolved from a European scholarly circle into a truly global academy, now comprising more than 600 members from over 50 countries. At the time CORLAS was founded, it had already been 55 years since the first female was admitted to study in Groningen, the Netherlands: Aletta Jacobs, who was also the first Dutch female to earn a doctoral degree in Medicine at the University of Groningen, in 1879 3 . Dida Dedering, the first CORLAS female member, attended the meeting in Copenhagen in 1948. The organization's historical records, however, show that nearly all presidents, general secretaries, treasurers and councillors were men for much of its first era. The role and representation of women in the CORLAS community reflect broader trends in academic medicine and surgical specialties. The fact that both the centennial meeting president and the next general secretary are woman reflects the importance of diversity and inclusion in the next era. While medicine, technology, and modes of communication have changed dramatically, the CORLAS has both embraced these changes and simultaneously remained committed to the idea that enduring scientific progress is best achieved through open dialogue among trusted colleagues and friends. This unique combination of academic excellence and collegial friendship has become one of the defining strengths of CORLAS.

The period from 2000 to 2025 illustrates this balance between continuity and renewal. Advances in genomics, translational medicine, cochlear implantation, robotics, artificial intelligence, and digital communication transformed both research and clinical practice. CORLAS embraced these developments while preserving the distinctive format that has characterized the society for a century: an annual meeting devoted to in-depth scientific discussion across the full spectrum of Otorhinolaryngology/Head and Neck Surgery.

During this period, meetings were held throughout Europe, Asia, North America, and South America. Major anniversaries were celebrated in Noordwijk (75 ^th^ anniversary, 2002), Moscow (80 ^th^ anniversary, 2006), and Bordeaux (90 ^th^ anniversary, 2016). The centennial meeting in Groningen is therefore more than another annual congress; it represents a symbolic return to the place where an enduring international community has been created.

The scientific scope of the Collegium has expanded considerably over time. Traditional strengths such as otology, hearing research and cochlear implantation remain central, while increasing attention has been given to head and neck oncology, rhinology, laryngology, sleep medicine, regenerative medicine, robotics, and artificial intelligence. The distinctive single-auditorium format continues to encourage interaction across subspecialties and generations, allowing all participants to share the same scientific experience from an interdisciplinary perspective.

Yet, the true uniqueness of CORLAS lies beyond its scientific program. For a century, annual meetings have intentionally combined academic exchange with opportunities for friendship, informal discussion, and cultural experiences. These interactions have fostered international collaborations, lifelong professional relationships, and a global network of scientific leaders. In many ways, the Collegium's founders understood an enduring truth: friendship is not separate from scientific progress but often one of its most powerful catalysts.

As our members gather in Groningen in 2026 − many arriving, just as their predecessors did a century ago, by train − they will return to the historical Academic Building where the first CORLAS session was held. Although the original Senate Room can no longer accommodate the number of members of the modern Collegium, the building itself offers enough space and it remains a tangible reminder of how a small gathering of visionary scholars grew into one of the most respected international societies in the field of Otorhinolaryngology.

The centennial celebration therefore marks more than the completion of a hundred-year history. It reaffirms the founding ideals established in Groningen in 1926: scientific excellence, international cooperation, and *Amicitiae Sacrum* . Those principles have guided CORLAS through its first century and will continue to shape its second.
